# The transmission dynamics of *Strongyloides stercoralis* and the impact of mass drug administration

**DOI:** 10.1098/rstb.2022.0442

**Published:** 2024-01-15

**Authors:** Benjamin S. Collyer, Roy Anderson

**Affiliations:** Department of Infectious Disease Epidemiology, Faculty of Medicine, Imperial College London, Praed Street, London W2 1PG, UK

**Keywords:** *Strongyloides stercoralis*, disease modelling, parasite

## Abstract

The epidemiology of *Strongyloides stercoralis* is briefly reviewed with an emphasis on cross section and longitudinal studies of infection prevalence stratified by age, performance of different diagnostic tools, mass drug administration (MDA) impact and estimates of key population parameters within the complex life cycle of the parasite that determine transmission intensity and response to control measures. The paucity of studies is highlighted, and gaps in current knowledge identified about the population biology of this very prevalent infection in tropical and sub-tropical regions around the world. A stochastic individual based stochastic model is described in part to highlight gaps in knowledge. The impact of repeated MDA is simulated to illustrate some aspects of transmission dynamics of this helminth infection. Specifically, the impact and bounce back times in either the intervals between treatment rounds, or post the cessation of treatment, depend critically on the magnitude of two distinct components of the basic reproductive number *R*_0_. The absence of data on these key components is highlighted, as is the value of studies of longitudinal cohorts of people in regions of endemic infection post rounds of MDA to record how infection levels bounce back post treatment at individual and population levels of study.

This article is part of the Theo Murphy meeting issue ‘*Strongyloides*: omics to worm-free populations’.

## 1. Introduction

**Figure 1.**
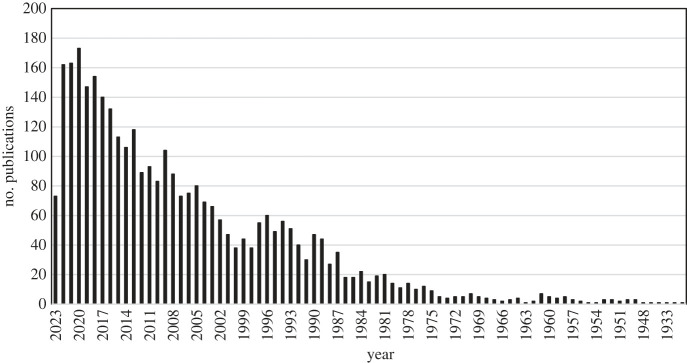
Number of publications per year on *S. stercoralis* NIH National Library of Medicine PubMed up to June 2023 (using search term *Strongyloides steracolis*).

Of the neglected tropical diseases one of the least investigated is the helminth parasite *Stongyloides stercoralis*, as reflected by the number of publications in the peer reviewed literature describing detailed epidemiological studies of transmission and health impact (figure 1). This parasite is thought to affect between 370 and 600 million people worldwide, and yet large gaps in knowledge exist about is epidemiology and public health significance [1] Recent analyses suggest between 161 and 284 million school-age children should be enrolled in deworming campaigns using ivermectin to substantially reduce the prevalence of strongyloidiasis [2]. These calculations were made to help endemic countries estimate the ivermectin supply needed for the implementation of control programmes for the control of strongyloidiasis.

The limited understanding of the epidemiology of this nematode infection is in part a consequence of difficulties in diagnosis via stool examination and the persistent nature of asymptomatic infection in many who acquire this parasite and serve as reservoirs of infection for others. Over the past decade, however, the seriousness of this infection as a cause of much morbidity and consequent mortality in immunocompromised individuals, has become more apparent and resulted in a surge of recent publications addressing how best to diagnose and treat, and where this infection is prevalent in tropical and warm temperate regions of the world. The National Institutes of Health in the United States via its PubMed citations for biomedical literature from MEDLINE, records a change from less than 20 publications per year on this parasite in 1986 to more than 160 in 2022 (figure 1). However, by comparison with other human helminth infections this remains a relatively small number of published peer reviewed studies.

With recent improvements in the sensitivity of new diagnostic methods to detect *S. stercoralis* larvae in human stool, a clearer picture is emerging of where this infection is common in tropical regions. Although a ‘gold standard’ diagnostic method has not as yet been defined by the World Health Organisation (WHO) [3], five methods are used in epidemiological surveys. These are a formol ether concentration technique (FECT), a spontaneous tube sedimentation technique, the Baermann concentration technique, an agar plate culture method, and a real-time polymerase chain reaction (RT-PCR). Recent studies in the Amhara region of northwest Ethiopia employing all these approaches on stool samples, for example, have revealed a high prevalence of infection in some regions of the country where other soil transmitted helminths (STHs) are also common, such as *Ascaris lumbricoides*, *Trichuris trichuria* and hookworm [4]. With what is assumed to be the most sensitive diagnostic, RT-PCR, the prevalence of infection approached 40% in a large sample of school-aged children. Similar levels of infection have been reported in Southeast Asia in countries such as Cambodia where prevalence in the provinces is typically around 20–40% [5]. More generally, recent work has attempted to produce global maps of infection distribution with some based on ecological niche modelling. The work of Fleitas *et al.* [6] and Eslahi *et al.* [1] has provided an important guide to where the new WHO strategy for the control of *S. stercoralis* infection involving mass drug administration (MDA) for school-aged children should be implemented. The authors of these studies highlight the various assumptions made in producing global maps and, concomitantly, the limited epidemiological data available employing sensitive diagnostic tools for many regions and countries. Aside from Africa, around the equatorial belt similar high prevalences of infection occur in Asia and South America [6]. One conclusion of this study is the apparent synchrony of the ecological niches of *S. stercoralis* and the human hookworms (*Necator americanus* and *Ancylostoma duodenale*). This is undoubtably related to the environmental conditions that determine the survival of free-living nematode larvae outside the human host.

The life cycle of *S. stercoralis* has some unusual properties that make control more difficult than is normally the case for other STH species [7]. The parasite is dioecious but the female worm within the human can reproduce parthenogenetically to produce eggs that hatch as either female or male larvae. A proportion of the eggs hatch in the intestine of the human host before leaving the host, with the remainder hatching in stool after it has been passed to the external environment. Male larvae develop in the free-living environment into free-living adult worms. Females on the other hand either develop into free-living adult females outside the host, or they can develop within the host into infectious L3 infectious larval stages and auto-infect the human host internally. If passed in the stool, they can externally infect the human host in a manner similar to the hookworm nematodes of humans. To add more complexity, mature adult male and females can mate sexually in the external environment to produce only one generation of L3 larvae that can infect the human host. In all this complexity the factors that determine the proportions of worms going down the different life cycle routes, and the magnitude of key population parameters, such as birth rates and adult and larval survival rates (life expectancies), are very poorly understood at present.

Two important features of this life cycle are of high importance in understanding the transmission dynamics of this infection; namely, the ability to auto reinfect and concomitantly for the infection to persist in the human host without reinfection for long periods of time involving many years despite a much shorter lifespan of any individual female adult worm in the host.

The sometimes profound pathology of this infection is associated with the autoinfective cycle with the larvae randomly migrating through tissue and the associated potential for lifelong infection once initially infected. A compromised immune system resulting in disseminated strongyloidiasis in humans has a very high case fatality rate, with 68.5% of 244 cases analysed in a systematic review having a fatal outcome [8]. Immunodeficiency is key to hyper-infection created by internal replication within the human host.

The population biology of the parasite is poorly understood at present and little published research exists on the transmission dynamics of *S. stercoralis* and the impact of MDA. This paper provides a brief summary of what is, and is not, known about the population biology of this nematode, details a simple mathematical model structure to explore the transmission dynamics under repeated MDA, and adapts an individual based stochastic model to examine possible outcomes of repeated MDA for parasite control. Model formulation of transmission and control helps to focus attention on what needs to be measured more precisely.

## 2. Epidemiological patterns and parameters—age prevalence profiles and reinfection studies

**Figure 2.**
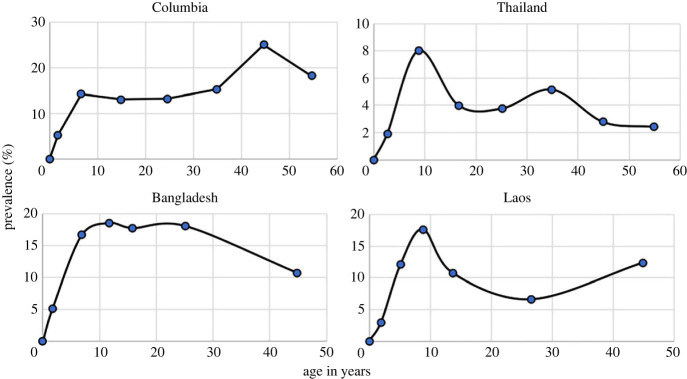
Age prevalence patterns of infection reaching a plateau with increasing age (from Conway *et al*. [9]).

**Figure 3.**
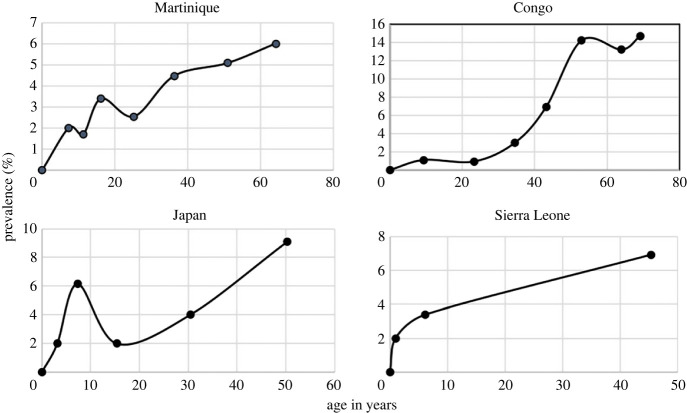
Age prevalence of infection with patterns increasing with age (from Conway *et al.* [9]).

For most STHs, the published literature records many examples of detailed epidemiological studies where age prevalence and age intensity of infection profiles stratified by various demographic variables such as age and gender are recorded. Of greater value are longitudinal studies where the impact of control interventions is assessed in cohorts of people stratified by age over repeated rounds of MDA treatment to assess rates of reinfection and hence measure the basic reproductive number *R*_0_ in defined settings. Few studies of this nature have been published to date for *S. stercoralis*, and most are based on prevalence records since the intensity if infection is difficult to measure given the limited sensitivity of current diagnostic tools and the complex life cycle where replication occurs within the infected host. Some of the more detailed prevalence examples with good sample sizes and stratified by age are recorded in figure 2 [9]. They reveal a mixed pattern of changes in prevalence by age, with some showing peaks in younger age groups while others record a more hookworm like pattern where infection levels increase with age (figure 3).

In all these studies, it should be noted that they do not use the most sensitive of the current diagnostic tools (RT-PCR) that was employed in the Ethiopian study of Jember *et al.* [4], and as such will underestimate true prevalence. To illustrate this point table 1 records estimates of prevalence by diagnostic used in the Ethiopian study of Jember *et al.* [4]. The large variation by method is very striking and suggests that most estimates in past studies in the published literature using other than the PCR method greatly underestimate prevalence.

**Table 1.** Diagnosis of *S. stercoralis* prevalence by stool examination using various diagnostic methods (from Jember *et al.* [4]).

diagnostic method	estimated prevalence of infection
formol ether concentration (FECT)	2.0
spontaneous tube sedimentation (STST)	4.0
Baermann concentration (BCT)	10.9
agar plate culture (APC)	28.8
real-time PCR (RT-PCR)	39.0

The most valuable information about the transmission dynamics of human helminth parasites in a defined locality, and the impact of control measures including MDA, is typically acquired from longitudinal studies of prevalence of infection in cohorts of people of mixed ages followed over time. Few such studies have been published to date hence the literature at present does not include papers that estimated rates of reinfection post treatment stratified by age and concomitantly, estimates of the magnitude of the basic reproductive number *R*_0_ [10].

Some of the most informative studies come from regions in which repeated rounds of MDA with the drug ivermectin (often combined with the use of albendazole or mebendazole to treat the other intestinal nematode infections) have been used to control onchocerciasis. A good example is the paper by Anselmi *et al.* [11] describing a study in Ecuador. The prevalence of infection based on the FECT (table 1) in children in regions where ivermectin was used regularly, fell from 6.8% in 1990 to 0% in 1996. By contrast, over the same period, in areas where ivermectin was not used the change observed was from 23.5% to 16.1%.

A larger study in Australia in an indigenous community also involving ivermectin MDA to only control *S. stercoralis*, showed great impact on the prevalence of infection based on serology when a high proportion of the community were treated. *Strongyloides* is endemic in many Australian Aboriginal and Torres Strait Islander communities with seroprevalence ranging from 35% to 60%. *Strongyloides stercoralis* seroprevalence reduced from 21% at baseline to 5% at month 6 after the first MDA. This reduction was sustained at month 12, falling to 2% at month 18 after a second ivermectin MDA round [12].

Turning to other sources of data on key parameters to aid in understanding the epidemiology and transmission of this parasite, information is even more limited with respect to adult worm life expectancy, developmental periods before maturity in the human host, female fecundity, density-dependent fecundity and larval life expectancy. Perhaps the most important of these are the developmental periods plus larval and adult worm life expectancy. Early self-infection studies suggest periods of between 17 and 60 days before larvae appear in the faeces post infection with L3 larvae [13]. The duration of infection post initial exposure can be very long given the autoinfective activity. A study of Australian Vietnam veterans found a *S. stercoralis* seropositivity rate of 11.6%, 40 years after the end of involvement in that conflict [14]. Another example demonstrated the persistence of infection over 65 years owing to the autoinfective route [15].

Individual adult worms may live for a few years, but data is only available from related infections in dogs. Egg production by female worms in the human host is low. The adult female only produces up to 40 eggs per day. Low and intermittent output of larvae is a thought to be a major factor for the low sensitivity of faecal testing, especially during the chronic phase of infection [7].

*Strongyloides stercoralis* larvae typically live for less than three weeks, even in soil under optimal conditions of moderate to high temperature and good moisture. Larvae die rapidly in unfavourable conditions, impeding faecal diagnostic tests unless stool examination occurs soon after release. As reviewed by Page *et al.* [7], this generation of filariform larvae are unable to develop into free-living adults, and if they fail to penetrate a human host, they die within two weeks or less in the free-living environment. The male and female free-living adult worms live for only a few days. This single free-living generation amplifies the number of infective filariform larvae contaminating the human environment. The duration of time in the environment of the free-living part of the parasite's life cycle is thought to be limited to three weeks or less.

No strong evidence exists at present to suggest the presence of an animal reservoir although further molecular epidemiological studies are required to eliminate this possibility.

From the limited data summarized above it is clear that the persistence of this infection, and the dominant aspect of its transmission dynamics, lies with the long-lived adult worm presence in the human host sustained by autoinfection, and not the persistent contamination of the host's environment with infected larvae. The short lived environmental contamination, boosted by one cycle of replication outside the human host (unique among human intestinal nematodes), results in spread between host, but effective treatment of the persistent human infection by ivermectin MDA seems the obvious control strategy linked with improved sanitation and clean water provision.

## 3. Mathematical models of transmission and control

**Figure 4.**
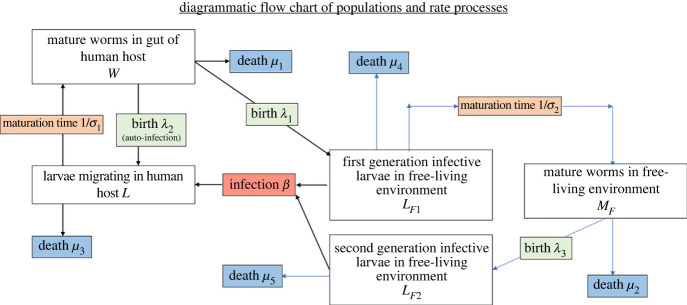
Flow chart of the key populations and population processes in the life cycle of *Strongyloides stercoralis*. (Online version in colour.)

To construct a model of the parasite's transmission dynamics, we have employed the framework of a soil transmitted nematode mathematical model of parasite transmission and MDA impact [10,16,17] and adapted an individual based stochastic STH transmission and treatment model [18–20] for the life cycle of *S. stercoralis* (figure 4).

In this adapted stochastic framework, each individual host, indexed by subscript *i*, harbours an integer number of larvae, *L_i_* and mature adult female worms, *W_i_*. *L_i_* and *W_i_* are stochastic processes whose dynamics, conditional on their rates, are birth–death processes. The rate of autoinfection is proportional to the host's current egg production rate, which includes a negative exponential density-dependent fecundity process [10], whose mechanism can be considered to be a product of the hosts immune system. The occurrence of widely disseminated infection throughout the human body when the immune system becomes compromised, well illustrates the importance of specific immunological responses in controlling parasite population size within the human host in those who are not compromised.

The rate of hosts acquiring new worms from the infectious material in the environment *β_i_* can be age dependent, and multiplied by a factor *g_i_* generated from a gamma distribution with mean 1 and shape parameter, *k*, which produces negative binomially distributed new infections from the environment, creating heterogeneous worm burdens.

The infectious reservoir consists of first- and second-generation filariform larvae in the free-living environment, whose densities are denoted *L_F_*_1,2_. The first-generation filariform larvae, which are excreted by the hosts at a rate proportional to the host's egg production rate, are infective and mature into mature adult sexually reproducing worms *M_F_*, whose offspring are the second-generation filariform larvae. The rates determining the dynamics of stochastic processes for the within host processes are given in table 2, and parameters are described in table 3. *L_F_*_1,2_ and *M_F_* are concentrations of larvae in the environment and are modelled by the ordinary differential equations:

3.1 $$\frac{dL_{F1}}{dt}=\underset{i}{\sum }\lambda_{1}W_{i}\exp(-\gamma W_{i})-(\mu_{4}+\sigma_{2})L_{F1},$$

3.2 $$\frac{dM_{F}}{dt}=\sigma_{2}L_{F1}-\mu_{2}M_{F}$$

3.3 $$\mathrm{and}\;\frac{dL_{F2}}{dt}=\lambda_{3}M_{F}\left(1-\exp\left(-\frac{M_{F}}{2}\right)\right)-\mu_{5}L_{F2}.$$

**Table 2.** Summary of the stochastic transmission process in each host.

process		rate
infection of free-living L3	*L_i_* → *L_i_* + 1	*β_i_g_i_*(*L_F_*_1_ + *L_F_*_2_)
autoinfection	*L_i_* → *L_i_* + 1	*λ*_2_*W_i_*exp( − *γW_i_*)
adult worm death	*W_i_* → *W_i_* − 1	*μ*_1_*W_i_*
larvae maturity	$\begin{array}{rl} & L_{i}\to L_{i}-\;1\\ & W_{i}\to W_{i}+\;1 \end{array}$	*σ*_1_*L_i_*

**Table 3.** Summary of parameter values.

parameter	description	value	source
*β_i_*	age dependent contact rate of individual host	determined by fitted *R_F_*	
*γ*	strength of within host antifecundity. The parameter *z* = e^−*γ*^ in equation (3.1)	0.02	assumed to be similar to *hookworm*
*λ*_1_	excreted egg production rate per female adult	40.0 d^−1^	[7]
*λ*_2_	within host production rate per female adult	determined by *R_I_*	
*λ*_3_	free-living egg production per female adult	40.0 d^−1^	assumed to be similar to within host egg production
*μ*_1_	death rate of adult female worms within host	1 yr^−1^	assumed similar to STH species
*μ*_2_	death rate of free-living adult worms	122 yr^−1^	[7]
*μ*_3_	death rate of larvae within human host	0	assumed to be zero (all larvae mature) as unknown
*μ*_4_	death rate of first-generation larvae	25 yr^−1^	[7]
*μ*_5_	death rate of second-generation larvae	25 yr^−1^	[7]
*σ*_1_	within host rate of maturation of immature larvae into reproductive adult females	6 yr^−1^	[7]
*σ*_2_	free-living larvae maturation rate	52 yr^−1^	assumed average maturation period of one week
*k*	shape parameter of gamma distributed risk, *g_i_*	0.01–0.11	fit to data

**Figure 5.**
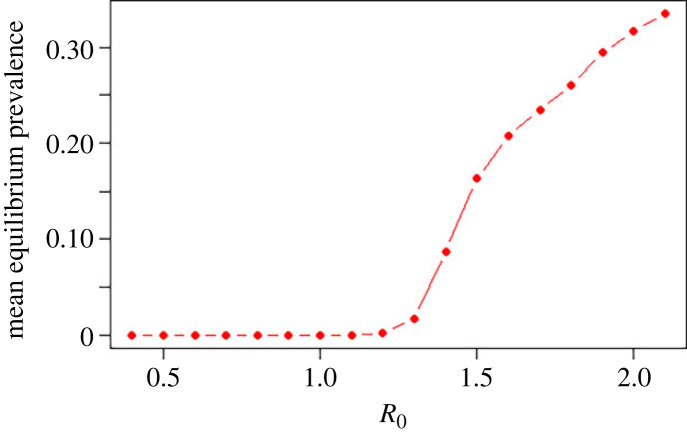
The mean equilibrium prevalence as a function of *R*_0_, with *R_I_* = *R_F_* = *R*_0_/2, *k* = 0.1. The estimates are generated from 50 time points after the system has reached an equilibrium, and averaged over 200 independent runs. (Online version in colour.)

The first term on the right-hand side of equation (3.1) accounts for the density-dependent probability of a first-generation female worm being fertilized by a male worm (the mating probability assuming worms are polygamous [17]). A diagrammatic flow chart of the processes is depicted in figure 5. We present results from this model without accounting for diagnostic methods, as the relationship between *S. stercoralis* infection and detection is as the discussed earlier variable depending on the diagnostic employed (table 1).

To illustrate the ability of the model to capture empirical epidemiological patterns, we have used an approximate Bayesian computation algorithm to fit the set of parameters {*R_I_*, *R_F_*, *k*} and relative age dependent contract rates to cross-sectional data collected in Cambodia [5]. We used the age aggregated prevalence as the summary statistic, non-informative priors and the Lenormand self-adaptive sequential algorithm to estimate the posterior distribution of the parameters [21,22].

## 4. Results

This model contains many parameters for which the current literature does not contain precise estimates. In their absence, we make assumptions based on our current limited understanding and explore the different behaviours that the model can exhibit. This stochastic transmission model resembles a class of models termed household or patch models, where a defining feature is the existence of two levels of mixing. In such models there are two different possible threshold quantities which determine the possibility for the system to reach an endemic equilibrium [23,24]. The first, *R*_0_, which, as is conventional for models of macro-parasitic disease transmission, is defined as the average number of offspring that survive to reach the reproductive adult female stage in the human host produced by a single adult female over the course of its lifetime in the absence of density-dependent processes (rather than the number of secondary infections of hosts). The second threshold quantity, often denoted *R_h_* in the household model literature, is the expected number of secondary infected hosts created by a single infected host over the course of their infectious period, and is more challenging to calculate for this model because infection within a host cannot be treated as a simple branching process.

Informally, we find *R*_0_ first by producing mean field equations for the stochastic dynamics. This can be achieved by define the mean quantities $M=\left\langle W_{i}\right\rangle$, $L=\left\langle L_{i}\right\rangle$, $\beta =\left\langle \beta_{i}\right\rangle$ and assuming that the distribution of worm burdens among hosts has a negative distribution, with aggregation parameter *k*, in order to calculate the mean egg production. This procedure [10], leads us to mean field equations:

4.1 $$\begin{array}{r} \frac{dL_{I}}{dt}=\;\beta (L_{F1}\;+\;L_{F2})+\lambda_{2}M{\left(1+M(1-z)/k\right)}^{-(k+1)}-(\mu_{3}+\sigma_{1})L_{I}, \end{array}$$

4.2 $$\frac{dM}{dt}=\sigma_{1}L_{I}-\mu_{1}M,$$

4.3 $$\frac{dL_{F1}}{dt}=\lambda_{1}M{\left(1+M(1-z)/k\right)}^{-(k+1)}-(\mu_{4}+\sigma_{2})L_{F1},$$

4.4 $$\frac{dM_{F}}{dt}=\sigma_{2}L_{F1}-\mu_{2}M_{F}$$

4.5 $$\mathrm{and}\;\frac{dL_{F2}}{dt}=\lambda_{3}M_{F}(1-e^{-M_{F}/2})-\mu_{5}L_{F2},$$

where *z* = e^−*γ*^. *R*_0_ can then be found as the largest eigenvalue of the next generation matrix for the system linearized around the worm-free equilibrium to be

4.6 $$R_{0}=\frac{1}{\mu_{1}}\left(\frac{\lambda_{2}\sigma_{1}}{\left(\mu_{3}+\sigma_{1}\right)}+\frac{\beta \lambda_{1}}{\left(\mu_{4}+\sigma_{2}\right)}\left(1+\frac{\lambda_{3}\sigma_{2}}{\mu_{2}\mu_{5}}\right)\right).$$

*R*_0_ is the sum of two terms, which can be considered to be the basic reproduction number of the free-living infection routes $R_{F}=\lambda_{1}\beta (1+(\lambda_{3}\sigma_{2}/\mu_{2}\mu_{5}))/\mu_{1}(\sigma_{2}+\mu_{4})$, and the basic reproduction number of the auto-infection route *R_I_* = *λ*_2_*σ*_1_/*μ*_1_(*μ*_3_ + *σ*_1_). We note that the small effect of the density-dependent sexual mating in the free-living infection route, which creates a less than 1 probability of female adults finding a mate, results in a critical *R*_0_ value that will typically be slightly larger than 1 [10]. The equilibrium prevalence of the stochastic model as function of *R*_0_ is displayed in figure 5. As is the case in all epidemic models, elimination of transmission will occur if an intervention is able to modify the parameters to reduce *R*_0_ to below its critical value for sustaining transmission.

**Figure 6.**
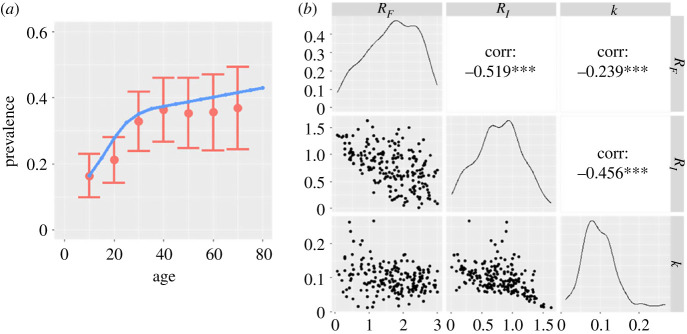
Fit of the stochastic model to age aggregated data [5]: (*a*) age aggregated prevalence, (*b*) prevalence as a function of *R*_0_, with *R_I_* = *R_F_* = *R*_0_/2, derived from numerical studies of the stochastic model. *** signifies the Pearson correlation coefficient is statistically significant at the *p*-value <0.001 level. (Online version in colour.)

The approximate Bayesian computation parameter fitted to the Cambodian prevalence data is shown in figure 6. Figure 6*a* displays the age profile of the fit to the data, while figure 6*b* displays the marginal and pairwise posterior distribution of {*R_I_*, *R_F_*, *k*}. The produced posterior distribution incorporates a large range of anticorrelated {*R_I_*, *R_F_*} pairs, illustrating the difficulty in inferring parameters well from baseline prevalence data alone.

To demonstrate the wide range of behaviours the model exhibits, we have selected two parameter sets from the posterior parameter distribution, chosen such that the free-living reproduction number is less than 1, and the internal greater than 1, and vice versa.

**Figure 7.**
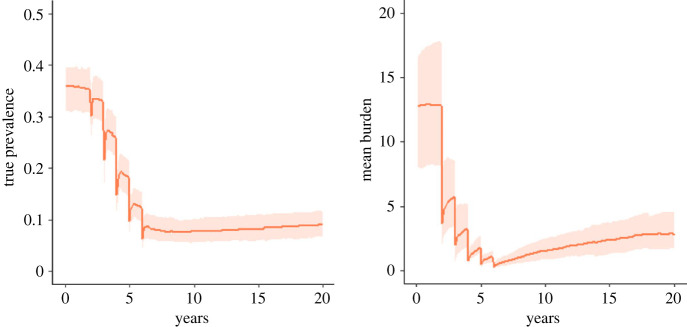
The true prevalence and mean burden across the total population following five rounds of annual mass treatment as predicted by the stochastic model. The orange line shows the mean model behaviour for *R_F_* = 0.9, *R_I_* = 1.6, *k* = 0.01 and the shaded area shows 95% of the distribution of outcomes. (Online version in colour.)

If the free-living reproduction number is smaller than 1, and the internal greater than 1, then we can expect the prevalence to respond much more slowly to perturbations from the equilibrium state, such as that induced by mass deworming treatment, because person-to-person transmission is relatively less likely (figure 7). These dynamics can result in long lasting, low prevalence that persists over many years, despite the burden increasing within infected individuals through the autoinfection route. In this regime, new infections in children are relatively rare, with the risk of new infection increasing slowly with age.

**Figure 8.**
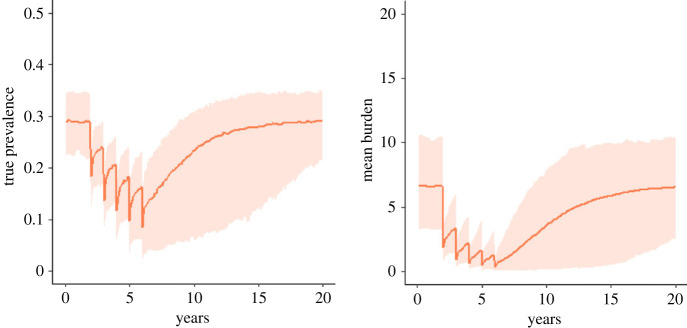
The true prevalence and mean burden across the total population following five rounds of annual mass treatment as predicted by the stochastic model. The orange line shows the mean model behaviour for *R_F_* = 2.3, *R_I_* = 0.5, *k* = 0.1 and the shaded area shows 95% of the distribution of outcomes. (Online version in colour.)

Conversely, if the external reproductive number is greater than 1 and the internal reproductive number is less than 1, then much faster returns to the endemic equilibrium state can be expected. In this regime, the transmission dynamics will be much closer to those exhibited by other helminth species (figure 8). The control of infection in such cases is much more challenging, as seen in the efforts to control, for example, schistosomiasis and STHs.

**Figure 9.**
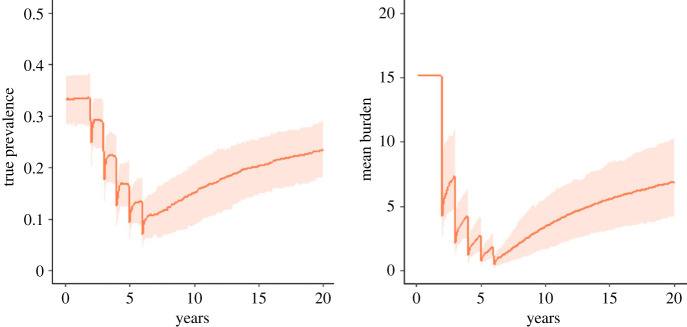
*R_F_* = 1.4 *R_I_* = 1.4, *k* = 0.05. The mean burden and true prevalence following five rounds of mass treatment as predicted by the stochastic model. The shaded areas show the range of outcomes generated by the stochastic model for a fixed parameter set. (Online version in colour.)

If both reproduction numbers are greater than 1 (figure 9), then infection will spread rapidly and an individual's worm burden can grow with speed even when their infection risk from free-living infective larvae is small.

## 5. Discussion

Model based analyses of the transmission dynamics of *S. stercoralis* show clearly that the frequency of MDA treatments in areas of endemic infection required to bring prevalence to very low levels will depend on the relative magnitudes of two key components of the basic reproductive number *R*_0_. This parameter measures transmission success in a defined habitat/environment and will vary location by location depending on cultural, human behavioural and climatic conditions. The latter influence the survival characteristics of the L3 infective larvae and hence the success of the fee living stages in transmitting infection from one person to another in a household or village.

These two components measure average reproductive success within the human host owing to the auto-infective route and the production of infective stages that pass to the exterior, while the second component measures transmission success in the external environment through one round of sexual reproduction and hence multiplication of the L3 infective stages. It is the second component of the production of infective stages in the external environment that most influences the frequency of treatment needed. If the magnitude of this component is much greater than unity in value, then treatment frequency must be high such as twice a year or yearly. If it is less than the value of unity in a dry setting with low average temperatures, then the intervals between treatments can be much longer. In both cases, however, given the high prevalences of infection recorded in many areas of endemicity using the most sensitive diagnostic method, irrespective of the relative magnitudes of the two components high MDA coverage will be required and good longitudinal compliance by individuals to repeated rounds of treatment.

How will it be possible to measure these two components? The easiest way is to record infection bounce back times in cohorts of repeatedly treated people stratified by age. A second method is good age stratified prevalence and infection intensity data in large samples of people in a cross-sectional study, since age measures time and hence differences between age groups reflect not just age dependent exposure but also the time interval dependent force of infection arising from the infective stages in the free-living environment. In turn this gives some insight into the magnitude of the second component of *R*_0_. Using the most sensitive diagnostic tool (RT-PCR) many more epidemiological studies of this nature are required as a matter of urgency.

One of the advantages of mathematical model development is it helps define what parameters must be measured to improve understanding of transmission and control. The models described above do just that. For *S. stercoralis*, the needs are great in terms of parameter estimates and the relative importance of the two major components that measure overall transmission success. Further model development is possible to refine how each stage in the complex life cycle influences the magnitude of *R*_0_ and how various components influence dynamical behaviour in response to control measures. Sensitivity analyses can be performed to gain insight into which parameters are influential for ongoing transmission and deeper analytical study in connection to meta-population models will generate generalizable understanding of the dynamics. We stress that while so many of the parameters remain uncertain the model should not be used to produce specific forecasts, and caution should be exercised when interpretting results. Given the high prevalence of infection in many regions of the world, plus the risk of serious pathology especially in the immune-compromised, much greater attention needs to be focused on this infection by the neglected tropical disease research community.

## Data Availability

This article has no additional data.
